# P009: Epidemiology, microbiology and outcome of community-οnset gram-negative bacteremias in a Greek University Hospital

**DOI:** 10.1186/2047-2994-2-S1-P9

**Published:** 2013-06-20

**Authors:** A Gikas, G Charitaki, M Gkika, P Karakosta, A Christidou

**Affiliations:** 1 Ind.Diseases Dpt-Inf Control Unit, University Hospiatal of Heraklion, Heraklion, Greece; 2 Clinical Bacteriology, University Hospiatal of Heraklion, Heraklion, Greece

## Introduction

According to the new classification community-onset bacteremias (COB) are divided in healthcare-associated (HCAB) and community-acquired (CAB). The objective of this study was to apply this classification to a cross-sectional study of patients with COB and to determine differences between the two groups in terms of epidemiology, treatment and outcome.

## Methods

The study was conducted at the University Hospital of Heraklion, Greece, from March 2010 to November 2011. Patients were classified as HCAB or CABusing pre-defined selection criteria. Epidemiological, clinical and therapeutic characteristics, antimicrobial resistance and outcome were compared in both groups. The statistical analysis was performed using SPSS 19.0.

## Results

Αmong 145 patients with gram-negative COB, 83 (57.2%) had HCAB and 62 (42.8%) had CAB. The frequency of malignant tumors, renal insufficiency and dementia was higher in patients with HCAB than with CAB. In both groups *Escherichia coli* was the mostcommon causative agent but the prevalence of *Klebsiella pneumoniae* in HCAB was significantly higher than CAB (19.3% vs. 4.8%). Patients with HCAB had higher Charlson score and higher Pitt bacteremia score, less frequent administration of appropriate empirical antibiotic treatment and higher probability of death than patients with CAB.

The antimicrobial resistance in HCAB και CAB patients respectively, was found 27/83 (32.5%) vs. 4/62 (6.5%) (P<.001) to third-generation cephalosporins (3GC), 22/83 (26.5%) vs. 7/62 (11.3%) (P=.021) to aminoglycosides, 29/83 (34.9%) vs. 9/62 (14.5%) (P=.005) to quinolones. Bacteria that produced ESBL were 16/76 (21.1%) vs. 2/59 (3.4%) (P=.003), and carbapenem-resistant were 10/83 (12.0%) vs. 2/62 (3.2%) (P=.056) in HCAB και CAB patients respectively

## Conclusion

There are significant differences in the severity of underlying diseases, causative pathogens, antibiotic resistance, outcome and treatment between the two groups. In our region 3GC, aminoglycosides or fluoroquinolones are proposed as appropriate empirical treatment for patients with CAB, whereas in patients with HCAB carbapenems should be the initial therapy

## Disclosure of interest

None declared

